# Involvement of ethylene and polyamines biosynthesis and abdominal phloem tissues characters of wheat caryopsis during grain filling under stress conditions

**DOI:** 10.1038/srep46020

**Published:** 2017-04-06

**Authors:** Weibing Yang, Yanxia Li, Yanping Yin, Zhilie Qin, Mengjing Zheng, Jin Chen, Yongli Luo, Dangwei Pang, Wenwen Jiang, Yong Li, Zhenlin Wang

**Affiliations:** 1 State Key Laboratory of Crop Biology, Ministry of Science and Technology, Shandong Agricultural University, Tai’an 271018, Shandong, China; 2 Beijing Academy of Agricultural and Forestry Sciences, Beijing Engineering Research Center for Hybrid Wheat. Beijing, 100097, China

## Abstract

Severe water deficit (SD) severely limited the photo-assimilate supply during the grain-filling stages. Although the ethylene and polyamines (PAs) have been identified as important signaling molecules involved in stress tolerance, it is yet unclear how 1-Aminocylopropane-1-carboxylic acid (ACC) and PA biosynthesis involving wheat abdominal phloem characters mitigate SD-induced filling inhibition. The results obtained indicated that the SD down-regulated the *TaSUT1* expression and decreased the activities of sucrose synthase (*SuSase*, EC2.4.1.13), ADP glucose pyrophosphorylase (*AGPase*, EC2.7.7.27), soluble starch synthase (*SSSase*, EC2.4.1.21), then substantially limited grain filling. As a result, increased ACC and putrescine (Put) concentrations and their biosynthesis-related gene expression reduced spermidine (Spd) biosynthesis under SD condition. And, the ACC and PA biosynthesis in inferior grains was more sensitive to SD than that in superior grains. Intermediary cells (ICs) of caryopsis emerged prematurely under SD to compensate for the weakened photo-assimilate transport functions of sieve elements (SEs). Finally, plasmolysis and nuclear chromatin condensation of phloem parenchyma cells (PPC) and membrane degradation of SEs, as well as the decreased ATP*ase* activity on plasma membranes of ICs and PPC at the later filling stage under SD were responsible for the considerably decreased weight of inferior grains.

Wheat (*Triticum aestivum* L.) grains are sensitive to a variety of environmental stresses throughout their growth period, such as salt, extreme temperatures, and drought^1^,^2^, which affect the crop production. Wheat crops need adequate water to reach their yield potential, and the lack of soil moisture during critical growth stages, especially grain filling, negatively affects productivity^3^,^4^. Adverse environmental stress caused by drought usually shortens the grain-filling period and reduces the grain-filling rate, which reduces grain weight^5^,^6^. Plants possess efficient defence mechanisms to overcome environmental stresses^7^. Polyamines (PAs) and ethylene may function as stress messengers involved in the responses of grain development to drought^8^,^9^,^10^.

Various stresses promote ethylene production in different plant tissues^11^,^12^. Drought-induced overproduction of ethylene has been closely associated with a reduction of grain weight in wheat^5^ and rice^13^. However, several reports suggested that water deficit decreases ethylene production^12^, and depends on the severity and duration of soil drying^5^. Application of ethylene inhibitors increased grain weight in wheat^5^ and improved dry matter partitioning and grain filling of basal rice kernels^14^, whereas ethephon (an ethylene-releasing agent) application showed the opposite effect. ACC concentration plays a decisive role in the ethylene evolution rate^15^ and its synthase gene (*ACS*) is present in all wheat tissues, especially during the early stages of spike development^16^. Based on the spatial position in wheat spikes, kernels at the base of the spikelets are regarded as superior grains, whereas the distal kernels are considered as inferior grains^17^. However, the responses of ethylene production, ACC concentration, and *ACS* expression in both grain types to severe water deficit (SD) and their relationship to filling, as well as the effects of chemical regulators, such as spermidine (Spd) and aminoethoxyvinylglycine (AVG), on grain filling are still unclear.

PAs, especially putrescine (Put), spermidine (Spd), and spermine (Spm), are involved in a wide range of biological processes, such as growth, development, and apoptosis^18^. Diamine Put biosynthesis has been previously described, the first steps of the pathway include ornithine or arginine decarboxylation, which is catalysed by ornithine decarboxylase (*ODC*) and arginine decarboxylase (*ADC*) in wheat^19^, respectively. The triamine Spd is synthesized via Spd synthase (*SPDS*) through the addition of an aminopropyl moiety to Put, donated by decarboxylated S-adenosylmethionine (dcSAM) formed by S-adenosylmethionine decarboxylase (*SAMDC*). PAs also exhibit anti-stress effects due to their acid neutralizing and antioxidant properties, and membrane and cell wall stabilizing abilities^20^. Since Put accumulation has been reported in leaf cells and protoplasts of oats in response to osmotic stress^21^, a similar increase has been exhibited in response to osmotic or drought stress in rice and other species^22^,^23^. Spd accumulate increased dramatically in cereal mesophyll protoplasts under osmotic stress^24^,^25^, whereas a reduction of Put or Spd levels has been observed in others under salt stress^26^. Put and Spd accumulation in rice in response to osmotic stress is possibly more dependent on stress intensity and duration^27^,^28^. However, the variations of PAs in grains of different kernel positions and their physiological function in grain filling under SD are unclear.

The vascular tissue of developing wheat caryopses consists of xylem and phloem (located immediately below the xylem). The phloem, composed of sieve elements (SEs), companion cells (CCs), intermediary cells (ICs), and phloem parenchyma cells (PPC), plays a central role in the long-distance transport of photo-assimilates^29^. Before assimilates enter the endosperm they traverse a short-distance between the terminal SEs of the vascular bundle and the aleurone layer^30^. A previous study showed that sucrose transporter (SUT) transcripts and SUT protein functional activity were located in the aleurone and nucellar cells of developing wheat caryopsis^31^. Naohiro^32^ reinforced the major role of *TaSUT1* in the post-phloem sugar transport pathway associated with the seed filling of wheat. Cytochemical studies provide a positive link between membrane transport processes and the activity of H^+^-pumping ATP*ase* on the plasmalemma^33^. Localization of ATP*ase* in SE-companion cell (SE-CC) complexes suggested that ATP*ase* activity is closely related to photo-assimilate accumulation, and ICs may play important roles in the long-distance transport at the later filling stage^34^. However, the SE-CC complex characteristics and *TaSUT1* expression, as well as ATP*ase* localization in phloem tissues with the grain development under SD remain unclear.

Overall, PA and ACC levels, and the phloem tissue characteristics of wheat caryopsis are significant in controlling the grain filling. In the present study, we investigated the effect SD on the different filling characters of superior and inferior at different grain filling stages. We elaborated the gene transcript level variations of encoding enzymes, which were involved in PA and ACC biosynthesis and SE-CC complex features in grain filling in response to SD. Exogenous Spd and AVG were used to verify the roles of Spd and ACC.

## Results

### Spd or AVG alleviate the SD-induced grain photosynthetic assimilation ability decreased

The SD had a significant influence on grain weight and starch content of both grain types (Table 1). SD decreased the weight of superior and inferior grains by 7.38 and 23.5% in JM22, respectively, and together with the more strongly decreased starch content in inferior grains. Likewise, SD showed a significant and negative effect on the active grain-filling period and mean grain-filling rate. However, application of Spd or AVG (ethylene biosynthesis inhibitors) significantly increased the grain weight and total starch content, indicating the positive role of Spd, and negative role of ACC in starch accumulation.

### Effects of SD on ethylene evolution rate, and ACC, Put, Spd production

**Figure 1:**
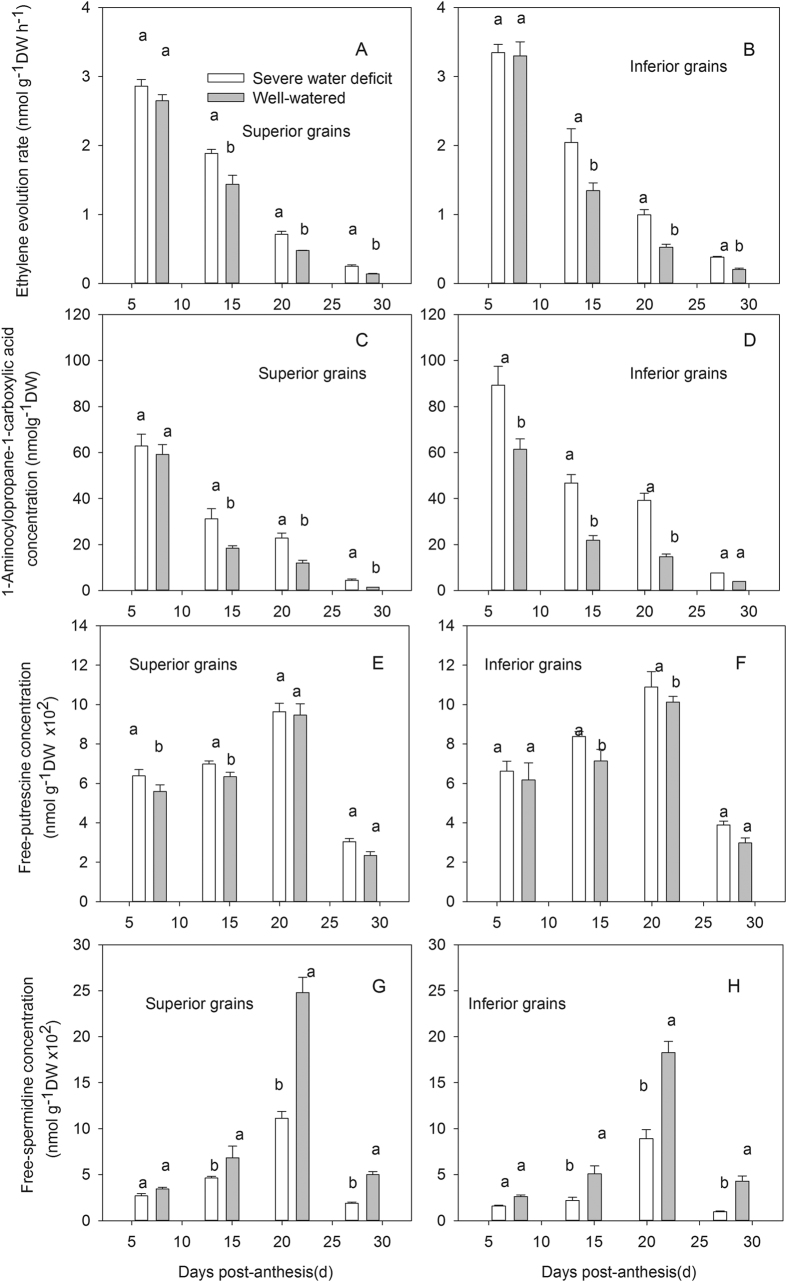
Post-anthesis severe water deficit effects on ethylene and polyamine content in wheat grains.

To confirm the roles of ethylene and PAs in SD-induced grain-filling inhibition, further experiment between ethylene emission and ACC concentration were performed on both grain types. As shown in Fig. 1, the ethylene emission or ACC concentration were high initially at the early grain-filling stage and sharply declined up to 28 DPA (Fig. 1A–D). Throughout the grain-filling period, the inferior grains showed a higher ethylene evolution rate and ACC concentration than the superior grains under WW or SD treatments. Thus, the ACC concentration of superior and inferior grains under SD increased markedly at 21 DPA, by 90 and 164%, respectively, and, the percentage increases of ethylene evolution rate in superior and inferior grains under SD were 40 and 100%, respectively. Also, the SD-induced ethylene production and ACC accumulation increase varied with grain types.

In contrast, the Spd concentration increased in both grain types during grain filling, and reached a peak at 21 DPA, then sharply decreased thereafter (Fig. 1G and H). Superior grains showed a higher Spd concentration than inferior during the grain-filling stage. Put in both superior and inferior grains showed a similar trend with Spd concentration, but inferior grains showed a higher Put concentration than superior (Fig. 1E and F). SD treatment significantly reduced the Spd concentration in both grain types compared with WW, and the percentage decrease was not significantly different between superior and inferior grains. Interestingly, the Put concentration was promoted by 1.04 and 7.3% in superior and inferior grains at 21 DPA under SD treatment.

### ACC and PA biosynthesis gene expression

**Figure 2:**
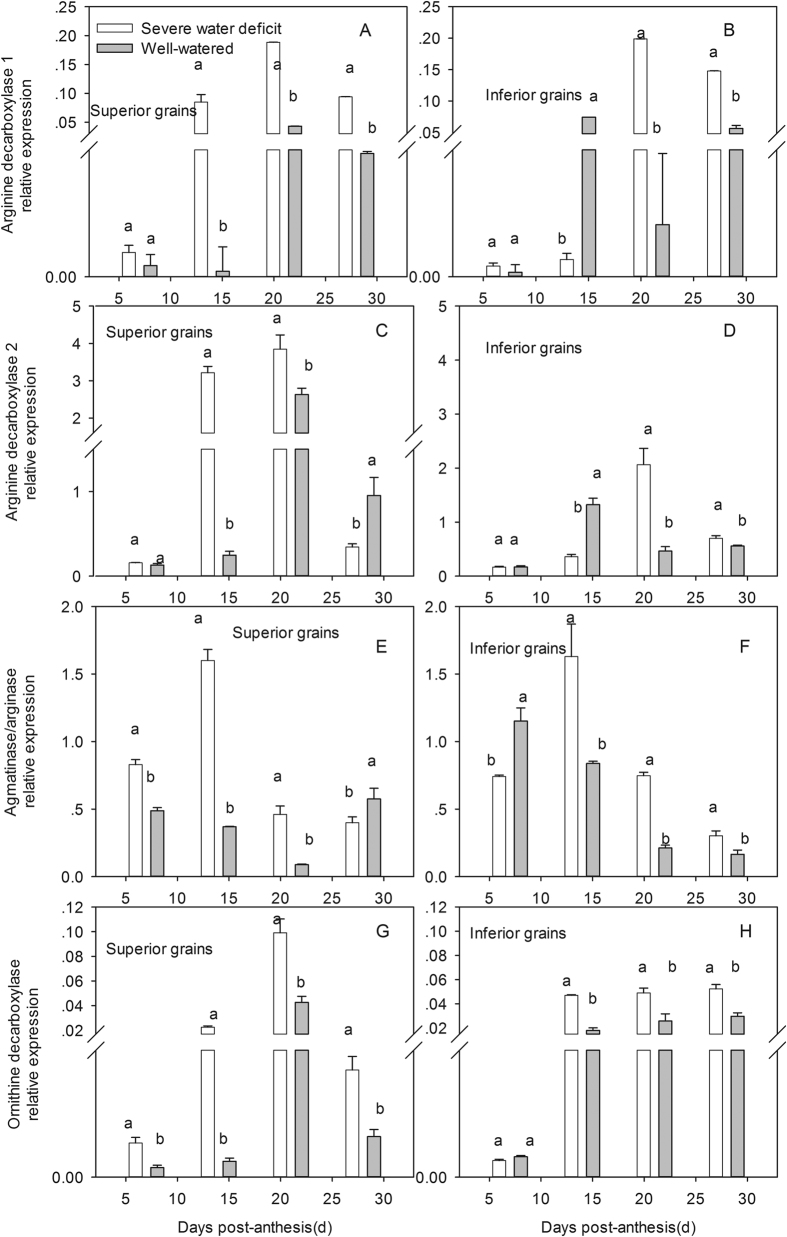
Post-anthesis severe water deficit effects on putrescine synthesis related gene expression in wheat grains.

Collectively, Put biosynthesis gene expression levels were low at the initial grain-filling stage and increased thereafter, and reached maximum expression levels at about 14 or 21 DPA, and then decreased until 28 DPA (Fig. 2A–H), which was highly consistent with the Put concentration variation and filling processes. Genes encoding enzymes involved in Put biosynthesis, including *ADC1, ADC2,* agmatinase/arginase, and *ODC*, were markedly increased by SD, except *ADC1* and *ADC2* in inferior grains at 14 DPA. Although, Put biosynthesis expression between superior and inferior grains varied with the grain development, to some extent, inferior grains showed a more sensitive of Put biosynthesis response to SD than superior ones.

**Figure 3:**
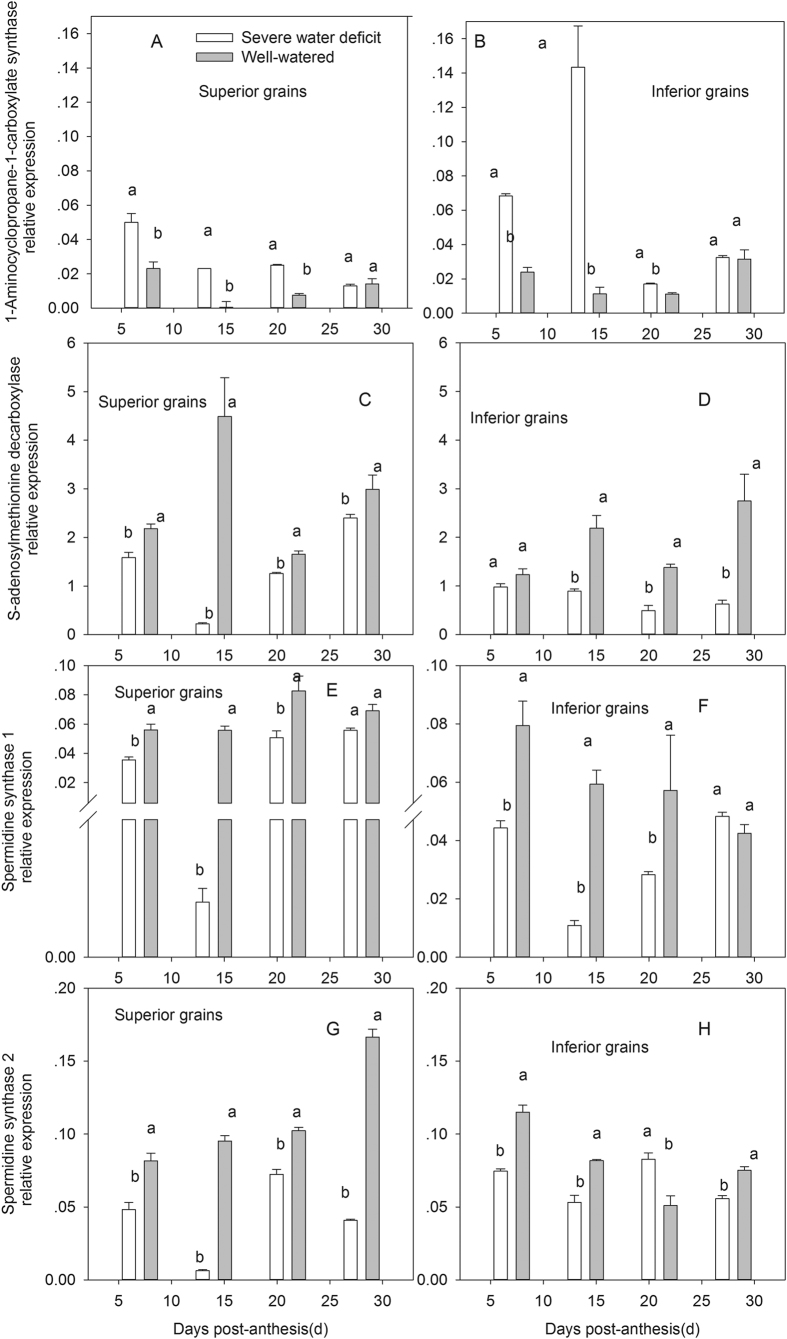
Post-anthesis severe water deficit effects on the 1-Aminocyclopropane-l-carboxylate synthase expression and spermidine synthesis related gene expression in wheat grains.

To verify the variations of ACC and PAs under SD determined by HLPC methods, expression analysis for genes encoding enzymes involved in ACC and PAs biosynthesis was performed. As shown in Fig. 3A,B, *ACS* expression level was significantly increased in response to SD at 7, 14, and 21 DPA, and was much higher in inferior than superior grains.

As shown in Fig. 3C–H, Spd biosynthesis genes exhibited the same trends as Spd concentration and grain development. *Spd1, Spd2*, and *SAMDC* expression in both grain types were strongly decreased by SD, and their expression levels in superior grains were higher than those in inferior ones, which were consistent with the difference of Spd concentrations between both grain types.

### Activities of key enzymes involved in sucrose to starch conversion

The effects of SD and chemicals on activities of key enzymes involved in sucrose to starch conversion in both grains types at 14 and 21 DPA were investigated (Table 2). SD obviously reduced the SuS*ase*, AGP*ase*, and SSS*ase* activity of both grain types. And, the application of Spd or AVG significantly increased the activities of key enzymes involved in sucrose to starch conversion in both gain types. Collectively, SD decreased the activities of *SuSase, AGPase*, and *SSSase* more significantly in inferior than superior grains. The percentage decrease of superior grains ranged from 2.3 to 33.6%, and 16.4 to 63.9% in inferior grains, indicating that the key enzymes involved in sucrose to starch conversion in inferior grains was responded more efficiently to SD than those in superior grains.

### Water soluble carbohydrates (WSC) content and *TaSUT1* expression

**Figure 4:**
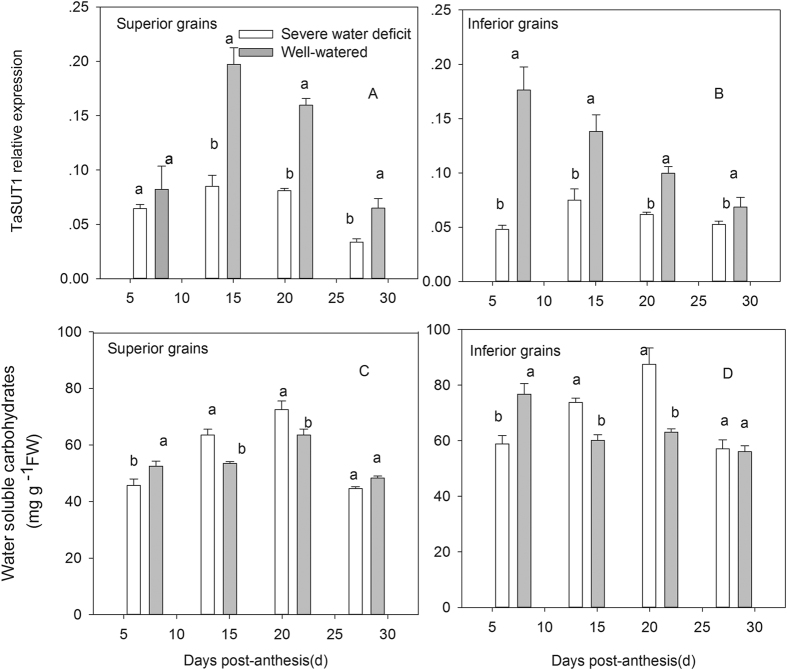
Post-anthesis severe water deficit effects on sucrose transporter (*TaSUT1*) expression and WSC concentration in superior and inferior grains. WW, well-watered; SD, severe water deficit; FW, Fresh weight.

Water soluble carbohydrates (WSC) content and *TaSUT1* expression of both grain types generally increased with grain development, reaching a maximum at the middle-filling stage, and then decreased gradually until filling was completed (Fig. 4A–D). SD significantly decreased the WSC content of both grain types at 7DPA, while significantly increased the WSC content of both grain types at 14 and 21 DPA. The difference of WSC content in both grain types between SD and WW was not significant at 28 DPA(Fig. 4C and D). *TaSUT1* had a major role in the post-phloem sugar transport pathway associated with grain filling. In the present study, SD apparently reduced the expression of *TaSUT1* (Fig. 4A and B). The WSC content and *TaSUT1* expression under SD varied with the grain types. The inferior grains had a higher WSC content and lower *TaSUT1* expression than superior grains during middle grain filling period at all conditions (SD and WW).

### Ultrastructure of the abdominal phloem of wheat caryopsis in response to SD

**Figure 5:**
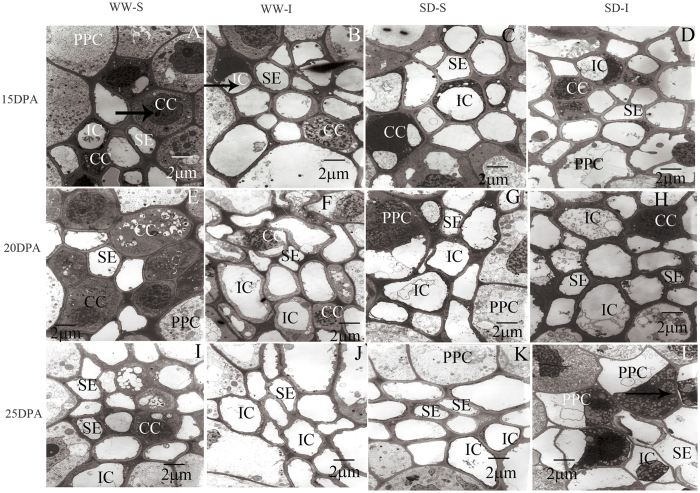
Transmission electron micrographs showing abdominal phloem tissue ultrastructure during wheat caryopsis in response to water deficit at different stages. WW, well-watered; SD, severe water deficit; S, superior grains; I, inferior grains; DPA, days post-anthesis; SEs, sieve elements; CCs, companion cells; ICs, intermediary cells; PPC, phloem parenchyma cells. (**A**), CCs with prominent nuclei (arrow) distributed in dense cytoplasm; (**B**), protoplasm (arrow) of ICs was distributed in the cell cavity; **L**, PPC (arrow) showed plasmolysis and nuclear chromatin condensation.

As the Fig. 5A–L showed TEMs of the ultrastructure of the abdominal phloem in wheat caryopsis at different filling stages under SD. Most of the SEs of the phloem were associated with CCs and had walls contiguous with those of the PPC. The superior grains under WW at 15 or 20 DPA showed structurally mature SEs of the abdominal phloem, and exhibited a clear lumen and plasma membrane, and CCs had prominent nuclei distributed in dense cytoplasm (Fig. 5A and E). At 15 and 20 DPA, ICs were found predominantly in SE-CC complexes. However, CCs were hardly observed in inferior grains under WW, or superior and inferior grains under SD. A few CCs were present in superior grains under WW, whereas were limited in inferior grains under WW, and superior and inferior grains under SD at 25 DPA. It is noteworthy that the PPC in inferior grains under SD showed plasmolysis and nuclear chromatin condensation, and the SEs exhibited membrane degradation at the later-filling stage, suggesting that PPC and SEs in inferior grains were more sensitive to SD than the superior ones.

### ATP*ase* activity in abdominal vascular phloem of inferior grains under SD

**Figure 6:**
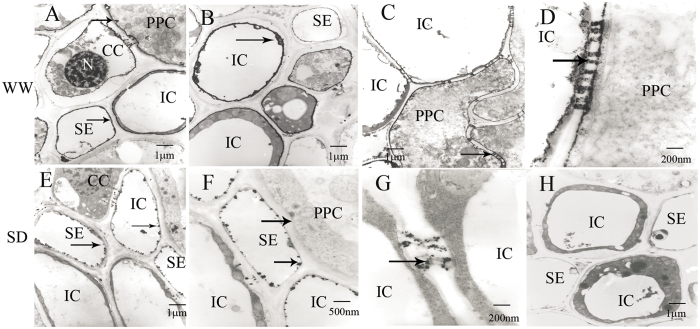
Transmission electron micrographs showing the localization of ATP*ase* activity on abdominal phloem tissues of inferior grains at 20 DPA. WW, well-watered; SD, severe water deficit; SEs, sieve elements; CCs, companion cells; ICs, intermediary cells; PPC, phloem parenchyma cells. (**A**), showed higher ATP*ase* activity (arrow) on plasma membrane of SEs and PPC; (**B**), showed higher ATP*ase* activity (arrow) on vacuole membrane of ICs; (**C**), showed higher ATP*ase* activity on plasma membrane of PPC; (**D**), plasmodesmata showed higher ATP*ase* activity (arrow); (**E**), showed lower ATP*ase* activity (arrow) on plasma membrane of ICs and SEs; (**F**), there was not active products of ATP*ase* on PPC (arrow); (**G**), plasmodesmata showed lower ATP*ase* activity (arrow); (**H**), control, no active products of ATP*ase* could be observed.

The present results indicated that the weight of inferior grains were more sensitive to SD than superior grains. Under WW, ATP*ase* activity in the plasma membrane of SE-CC complexes and ICs (Fig. 6A and B) and PPC (Fig. 6C) visualized by particles of lead phosphate precipitate was considerably increased than that of the control (Fig. 6H). The band of lead phosphate precipitate on the plasma membrane was intensified, widened, and became continuous as consequence of the precipitate increase. In addition, the plasmodesmata between ICs and PPC showed a high density of lead phosphate precipitate (Fig. 6D). Under SD, the plasma membrane of SEs and ICs (Fig. 6E and F) and the plasmodesmata between ICs and ICs (Fig. 6G) showed a lower density of lead phosphate precipitate. Unlike SEs and ICs, limited ATP*ase* activity was observed in the plasma membrane of CCs and PPC.

## Discussion

In the present study, SD sharply reduced the mean grain filling, active filling period, and starch content, which could partially lead to the decreased weight of both grain types. Furthermore, SD down-regulated *TaSUT1* expression, but increased the WSC content at 14 and 21 DPA. These alternations in WSC content and *TaSUT1* expression combined with the strongly decreased activities of SuS*ase*, AGP*ase*, and SSS*ase* under SD confirmed that the starch biosynthesis enzymes activities, rather than photo-assimilate supply, were a major constraint on grain filling at the middle-filling stage, which concurs with previous reports^15^,^35^. The results also indicated a large difference in the decrease of grain weight between different kernel positions of wheat spikelets^7^,^36^, 7.38 and 23.5% for superior and inferior grains, respectively. Nevertheless, few reports focused on the involvement of ACC and PA biosynthesis in the different responses of grain weight of superior and inferior to SD. SD generally down-regulated the gene expression of *Spd1, Spd2,* and *SAMDC*, and thus decreased the Spd concentrations. Spd possibly mediated the effect of SD on grain filling based on the positive relations between Spd and starch synthesis. Exogenous methylglyoxal-bis (an inhibitor of Spd and Spm synthesis) or exogenous Spd was used and properly proved the positive role of Spd on grain filling^35^,^36^. The higher percentage decreases of *SuSase, AGPase,* and *SSSase* activities, and Spd concentration in inferior grains than those in superior ones can sufficiently explain the different responses of grain types to SD. In the present study, Put exhibited a negative relationship to the activities of starch biosynthesis enzymes under SD, mainly due to excessive Put accumulation under SD that negatively affected *SuSase, AGP*ase, and *SSSase* activities, which was supported by previous research^37^,^38^. To verify the reliability of the Put concentration variation, which was determined by HLPC, the expression of Put biosynthesis genes were studied by qRT-PCR. As expected, SD generally up-regulated the expression of *ODC, ADC1, ADC2* and agmatinase/arginase genes, and again, confirmed that excessive Put accumulated in response to SD, which was supported by previous literature^21^,^39^. Put biosynthesis of inferior grains was more affected by SD than that of superior ones, which concurred with the higher decrease in weight of inferior grains than that of superior ones.

The mechanism by which Spd or Put facilitates grain filling has been proposed that Spd may partially replace calcium in maintaining membrane integrity by binding to phospholipid components of the membrane under stress conditions^40^. Spd and Spm biosynthesis play key roles in the grain-filling process of maize^41^ and rice^15^. In our experiment, we proved that a higher Spd concentration, caused by the application of exogenous Spd or AVG, play pivotal roles in grain filling through regulating the activities of key enzymes involved in the sucrose-to-starch conversion. It was noted that Put could activate antioxidant enzymes and elevate antioxidants, thereby controlling free radical generation and preventing membrane peroxidation and biomolecule denaturation, leading to improved seedling growth under salinity stress^42^,^43^. However, a mass Put accumulation is generally toxic to plants^44^, depending on the severity and duration of the stress^25^,^27^. The present results indicated that Put accumulation under SD negatively affected starch biosynthesis enzyme activities. This was consistent with research that indicated that increased Put concentrations negatively affected tobacco morphology, such as leaf wrinkling, chlorosis, necrosis, and reduced stem and root growth^45^. The effect of water deficit on ethylene production is unclear^32^, Some studies indicated that moderate soil drying decreased the ethylene evolution rate significantly, whereas SD showed an opposite effect^39^. Our results showed that SD significantly enhanced *ACS* expression level and thus ACC concentration and ethylene evolution rate, consistent with the possibility that ethylene production in wheat grains depends on the severity and duration of soil-drying^5^,^39^. The ethylene evolution rate and ACC concentration negatively correlated with the starch biosynthesis enzyme activities and grain-filling rate, confirming a negative role of ACC on grain filling. The higher percentage increase in ACC concentration of inferior grains than that of superior ones under SD resulted in a more significant weight reduction in inferior grains.

It has been suggested that plant hormones act either synergistically or antagonistically and it is the balance between promoting and inhibiting agents that ultimately determines the path of plant growth and development^46^. PAs (Spd and Spm) and ethylene share a biosynthetic precursor SAM, and the increase in Spd levels possibly affect the rates of ethylene biosynthesis^41^,^47^. In the present results, SD decreased Spd levels, but increased the ACC concentration, which combined with the positive relationship between grain filling and Spd/ACC, indicating that Spd and ACC exhibited an antagonistic relationship. Spd/ACC in inferior grains under SD decreased more than in superior grains, which was one of the physiological reasons for the more significant weight reduction of inferior grains. In summary, we speculated that the increase in ACC and Put concentration and the decrease in Spd concentration, and the balance between Spd and ACC mediated the reduction of the grain-filling rate under SD. Similar relationships have been observed in rice under severe water deficit^40^.

In developing wheat grains, photosynthate is transferred longitudinally along the phloem and then laterally into the endosperm cavity^29^,^30^. The abdominal phloem characteristics of wheat caryopsis play an important role in photosynthate transport^34^. So far, limited reports have focussed on the responses of abdominal phloem characteristics of wheat caryopsis to SD. In the present study, CCs in superior caryopsis under WW contained electron-dense protoplasm and nuclei under WW at the early- and middle-filling stage. However, CCs in both grain types under SD showed significant vacuolar structures at the initial-filling stage, indicating that the cytoskeleton was destroyed and the metabolism was seriously affected by SD^48^, which was similar to the reports where CCs showed small vacuolar structures and few mitochondria and endoplasmic reticulum in weak-light source leaves and fruits of nectarine trees^49^. Previous studies concluded that SEs function in transport at the early- and middle-grain filling stages, and ICs had the same structure as SEs when the protoplasm was dispersed in the cell cavity and were considered as special companion cells adapted to photo-assimilate transportation at the later-filling stage^34^. In the present study, the amount of ICs increased significantly with grain development under SD, indicating that the ICs emerged prematurely to compensate for the decreased photo-assimilate-transport functions of SEs. In addition, the WSC content and the *TaSUT1* expression level in superior and inferior grains at 28 DPA were significantly decreased when compared with those at 21DPA, and SD slightly decreased the WSC content in superior grains at 28 DPA. These results, combined with the variations in the structure and functions of abdominal phloem tissues of caryopsis, substantiated that the carbohydrate supply at the later-filling stage may be a major factor limiting grain filling. The PPC of SD-I showed plasmolysis and nuclear chromatin condensation, and the SEs exhibited membrane degradation characteristics. Based on the inferior caryopsis at 20 DPA under SD conditions, of which the plasma membranes of SEs and ICs and plasmodesmata between ICs and ICs showed a lower density of lead phosphate precipitate, and ATP*ase* activity was limited in the plasma membranes of CCs and PPC, we can conclude that the more sensitive responses of the phloem transport functions in inferior caryopsis under SD may lead to a higher weight reduction of inferior than superior grains.

In summary, our study revealed the roles of Put, 1-Aminocylopropane-1-carboxylic acid (ACC), and Spd, and Spd/ACC on mediating the effects of severe water deficit (SD) during grain filling. It would benefit wheat to show the physiological traits of higher Spd and Spd/ACC under SD. The higher weight decrease of inferior grains than superior grains in response to SD was mainly due to a more significant decrease in enzyme activities related to starch biosynthesis at the middle-filling stage, and a noticeable decline in the transport ability of abdominal phloem tissues of inferior grains at the later-filling stage.

## Materials and Methods

### Plant material and cultivation

The experiments were performed at the Experimental Station of Shandong Agricultural University from October 2011 to June 2012 growing seasons, Tai’an, China (36°18’ N, 117°13’ E). Jimai22 (JM22) is a multiple-spike cultivar with a low frequency of tertiary kernels in spikelets and more tillers, was grown in 150 cm deep cement tanks (width: 300 cm and length: 250 cm) in open field conditions. The tanks were isolated from each other by a ridge (30 cm wide) covered with cement. Each tank was filled with sandy loam soil, and the 0–20 cm soil layer contained 10.9 g total organic matter kg^−1^, 0.8 g total N kg^−1^, 78.98 mg available N kg^−1^, 28.49 mg available P_2_O_5_ kg^−1^, and 98.32 mg available K_2_O kg^−1^. The sowing date was the 8th October 2011, and plant density was adjusted to 180 plants m^−2^ at the three-leaf stage (GS13)^35^. Nitrogen (N; 195.6 g tank^−1^ as urea), phosphorus (P; 468.7 g tank^−1^ as single superphosphate), and potassium (K; 150 g tank^−1^ as KCl) were applied as a basal fertilizer, with N (195.6 g tank^−1^ as urea) being top-dressed at the jointing stage (GS31)^50^. There was no noticeable crop damage from weeds, insects, and diseases, and no special weather events during grain filling. Maximum and minimum air temperatures during the grain-filling stage were 33.3 and 7.8 °C, respectively.

### Experimental design, soil-drying treatments, and chemical applications

The experiment was design with 6 treatments [one cultivar, two levels of soil moisture, and three exogenous regulator treatments (two chemical regulators and the control)] and all trials was a randomized complete block with three replicates and total 18 tanks. The soil water content was maintained close to field capacity [soil water potential (wsoil) ranged from −0.02 to −0.025 MPa] by manual watering until 3–4 DPA when soil-drying treatments were initiated. The well-watered (WW) treatment was maintained at −0.02 to −0.025 MPa, and the SD treatment was maintained at −0.07 to −0.08 MPa. Soil water potential was monitored at 25- to 30-cm soil depth. Tension meters (Beijing, Zhongxi Co., China) were installed in each treatment and were recorded at 14:00 daily. When the reading dropped to a certain value, 0.04 and 0.02 m^3^ of water per plot were added to the WW and SD plants, respectively. A rain shelter consisting of a steel frame covered with plastic plates (90% light transmittance) was used to protect the plots from rain.

Starting at 1 DPA, 1 mM Spd, and 40 μM AVG (an inhibitor of ethylene biosynthesis by inhibiting ACC synthesis) (Aladdin, China) were applied to spikes in both WW and SD treatments by using an atomizer. The chemicals were applied daily for 4 d at a rate of 2 L per tank at each application, with 0.01% (v/v) Tween-20 (Fluka, Riedel-de-Haen, Germany) as a surfactant. All the solutions contained ethanol and Tween-20 at a final concentration of 0.1% (v/v) and 0.01% (v/v), respectively. The same volume of deionized water containing the same concentration of Tween-20 was applied to the controlled plants.

### Sampling

About 200 spikes that flowered on the same day were tagged for each experimental plot. From 7 days post anthesis (DPA), 20 labelled spikes of each plot were sampled at 6-d intervals until 28 DPA. The first and second basal grains on the spikelets were termed superior grains, whereas the third and fourth grains on the same spikelets were termed inferior grains^17^. Half sampled grains were immediately frozen in liquid nitrogen for about 30 min and kept at −75 °C and the remaining grains were dried at 70 °C to a constant weight. The materials used for the determination of all gene expression were from WW and SD plants without exogenous applications, while that used for activities of key enzymes determination were from all treatments (control and exogenous application). At maturity, 20 labelled spikes of each plot were sampled and used for the analysis of grain weight and starch content.

The grain filling process was fitted by the Richards’s growth equation (Equation 1)^51^


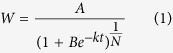


The mean grain-filling rate (G) was calculated by Equation 2


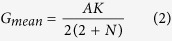


W, the grain weight (mg); A, the final grain weight (mg); t, days post-anthesis; B, k and N, coefficients determined by regression.

### Ethylene and ACC analysis

Ethylene emission from grains was determined according to Yang^5^ with modifications. Briefly, sampled grains were placed between two sheets of moist paper for 1 h at 27 °C in darkness to allow wound-induced ethylene production to subside. Between 30–40 grains were then transferred to 15 ml glass vials containing moist filter paper, and incubated in the dark for 12 h at 27 °C. A 1-ml gas sample was withdrawn through the seal with a gastight syringe and ethylene was assayed using a gas chromatograph (GC solution-2010 series, Japan) equipped with a Porapak Q column and flame ionization detector (FID).

The ACC concentration in the grains was determined according to Cheng and Lur^52^. Briefly, sampled grains were ground in liquid N_2_, and 0.1 g of the resulting powder was homogenized with 2 ml 95% ethanol at 80 °C for 15 min. Samples were then centrifuged at 4 000 × g for 15 min. The extraction was repeated twice and the supernatants were combined, dried in N_2_ and resuspended in 2 ml distilled water and 2 ml chloroform to remove lipids. Following partitioning, the chloroform fraction was discarded. After incubation, and 1 ml gas sample was with drawn from the tube headspace with a syringe. Ethylene evolved from ACC was assayed using gas chromatography as described in the previous paragraph.

### Extraction and quantification of Put and Spd

Free-Put and -Spd were estimated and quantified by HPLC (Waters 2998, USA) as described by Flores and Galston^53^. Briefly, 20–30 grains were ground in liquid N_2_, and resuspended in 5 ml 5% (v/v) perchloric acid. The samples were incubated at 4 °C for 2 h and then centrifuged at 10000 × g for 15 min. 1 ml aliquot of the supernatants and standard solutions of Put and Spd were dansylated at 37 °C for 1 h using 20 μl benzoyl chloride and then terminated by adding 2 ml saturated sodium chloride. Diethyl ether (2 ml) was added to the solution, removing 1 ml of the mixed and air-drying it. The sample was dissolved in methanol [1 ml, (60%, v/v)], mixing for 1 min, then 20 μl of the filtered sample was injected.

### Water soluble carbohydrates (WSC) content and enzyme assays

The levels of total WSC were determined by sequential extraction in 80% ethanol and water according to the reports^54^ followed by determination in the extract using the anthrone method of Yemm and Willis^55^. The starch content were determined as described previously^56^. Enzyme extraction and the enzyme assays were according to the method described by Ranwala^57^ and Nakamura^58^. Briefly, a 1 g sample was homogenized in an ice-cold mortar in 8 ml of 50 m*M* HEPES-sodium hydroxide (NaOH) (pH 7.5) containing 50 m*M* HEPES, 5 m*M* EDTA, 1 mM dithiothreitol (DTT), 2 m*M* KCl, and 1% polyvinylpyrrolidone (PVP). The homogenate was centrifuged at 10000 × g at 0–4 °C for 15 min and the supernatant was used.

### RNA extraction, cDNA biosynthesis, and quantitative real-time PCR

RNA extraction was performed using TRIzol reagent. Genes were measured by ABI PRISM 7700. Primer sequences used to amplify wheat PA and ACC biosynthesis and TaSUT1 genes are as follows:

**Ornithine decarboxylase (ODC):** GTGCGTGGAGGTGATAGGC, CATGGGGATGCAGCTGAG;

**Arginine decarboxylase 1(ADC1):** CACCAAGATACCAGGCCACT, GTGGAAGTGCAGCAACTTGA;

**Arginine decarboxylase 2(ADC2):** AGGAGGAGGAGCTCGACATT, GCCGAACTTGCCCTTCTC;

**Agmatinase:** GGGAAGAGATTTGGTGTGGA, TCACACCTTCCCCAAGTTTC;

**S-adenosylmethionine decarboxylase(SAMDC):** GCGTCCTCATCTACCAGAGC, CTTGCCTTCCTTGACCAGAG;

**Spermidine synthase 1(Spd1):** TGATTCAGGACATGCTTTCG, CCCAATTGCACCACTAGGAT;

**Spermidine synthase 1(Spd2):** CACACACATCTAATCCAG, ATCCAATGACACCACT;

**1-Aminocyclopropane-l-carboxylate synthase (ACS):** TCCGTCACCATACTACCC, CCCTTACTCCTCGCTTC;

**Sucrose transporter (TaSUT1):** TATTCCTGCTGCCCAAGAT, CACACAAGTCACAACCCAA;

**Actin:** AGAGAGAAAATGACCCAGA, CCAAACGAAGGATAGCA.

### Transmission electron microscopy

Features of abdominal phloem tissues of wheat caryopsis at 15, 20, and 25 DPA were investigated by transmission electron microscopy (TEM). Sections (80–90 nm thick) were cut with an LKB 2088 ultracut ultramicrotome (Reichert Supernova, Leica, Germany), Transverse sections of phloem tissues were viewed using a TEM (JEM-1200EX, Hitachi, Japan).

### Cytochemical localization of ATP*ase* activity in phloem tissues

The small pieces of vascular tissue were normally fixed in a mixture of 2% glutaraldehyde and 4% formaldehyde in 50 m*M* cacodylate buffer (pH 7.2) for 2 h at 4 °C. After fixation, tissues were washed in 50 m*M* cacodylate buffer (pH 7.2) three times, and then rinsed with a 50 m*M* Tris-maleate buffer (pH 7.2) (3 × 30 min). The tissues were incubated in a medium containing 2 m*M* ATP, 3 m*M* Pb (NO_3_)_2_, 5 m*M* MgSO_4_, and 60 m*M* Tris-maleate buffer (pH 7.2) at 23 °C for 2 h. For the control, 10 m*M* sodium fluoride (NaF) was added to the incubation medium. All tissues were subjected to a 2–3 h post-incubation rinse by a 50 m*M* Tris-maleate buffer (pH 7.2) three times, and then 50 m*M* cacodylate buffer (pH 7.2) (3 × 30 min), followed by an overnight post-fixation in 1% OsO_4_ in 50 m*M* cacodylate buffer (pH 7.2) at 2–4 °C. After post-fixation, tissues were rinsed (3 × 10 min) in a cacodylate buffer, dehydrated in a graded ethanol series, and infiltrated with resin. Transverse sections of phloem tissues were viewed, without post-staining, using a TEM (JEM-1200EX, Hitachi, Japan).

### Statistical analysis

Analysis of variance (ANOVA) was performed with PASW software version 18.0. Data from each sampling date were analysed separately. Means were tested by Tukey’s multiple comparison test at the *p* < 0.05 level.

## Additional Information

**How to cite this article:** Yang, W. *et al*. Involvement of ethylene and polyamines biosynthesis and abdominal phloem tissues characters of wheat caryopsis during grain filling under stress conditions. *Sci. Rep.* **7**, 46020; doi: 10.1038/srep46020 (2017).

**Publisher's note:** Springer Nature remains neutral with regard to jurisdictional claims in published maps and institutional affiliations.

## Supplementary Material

Supplementary Information

Supplementary Data

## Figures and Tables

**Table 1:** Chemicals and post-anthesis severe water deficit effect on grain weight (mg) and starch contents (%) at maturity, active grain filling period (d) and mean grain filling rate (mg per kernel d^−1^).

		Grain weight (mg)		Starch content (%)		Active grain-filling period (d)		Mean grain-filling rate (mg grain^−1^ d^−1^)	
Treatment		S	I	S	I	S	I	S	I
WW	Control	46.71 ± 0.37c	40.31 ± 0.15b	72.85 ± 0.62bc	55.16 ± 0.82c	31.3 ± 0.36a	30.15 ± 0.34bc	1.7 ± 0.02a	1.31 ± 0.01abc
	Spd	49.2 ± 0.65ab	43.75 ± 0.57a	79.18 ± 1.04a	58.67 ± 0.33ab	33.19 ± 0.88a	33.3 ± 1.16a	1.7 ± 0.06a	1.33 ± 0.03abc
	AVG	49.75 ± 1.22a	43.44 ± 0.71a	79.71 ± 0.78a	59.72 ± 0.95a	33.3 ± 1.62a	32.5 ± 0.59ab	1.7 ± 0.01ab	1.55 ± 0.02a
SD	Control	43.26 ± 0.46d	30.82 ± 0.19d	69.21 ± 0.65c	47.25 ± 0.33d	28.1 ± 0.96b	28.3 ± 1.1c	1.43 ± 0.06bc	1.18 ± 0.16bc
	Spd	46.55 ± 1.28c	35.72 ± 0.95c	74.81 ± 0.57b	56.38 ± 0.19bc	30.77 ± 0.52ab	30.2 ± 0.74bc	1.38 ± 0.07c	1.04 ± 0.05c
	AVG	46.89 ± 1.32bc	34.56 ± 0.77c	76.07 ± 1.49ab	57.53 ± 0.83abc	30.8 ± 0.23a	30.9 ± 0.91abc	1.65 ± 0.01ab	1.38 ± 0.04ab
P-value	Water treatment (W)	0.0064	0.0001	0.0002	0.0001	0.0001	0.0008	0.0008	0.0032
	Chemical treatment (C)	0.0130	0.0003	0.0001	0.0001	0.0022	0.0017	0.0838	0.0032
	W × C	0.7150	0.6414	0.8819	0.0024	0.7761	0.3798	0.0519	0.4498

Means (*n = *3) within a column with the same letter are not significantly different at *P* < 0.05. WW, well-watered; SD, severe water deficit; AVG, Aminoethoxyvinylglycine; Spd, Spermidine;

S, superior grains; I, inferior grains.

**Table 2:** Chemicals and post-anthesis severe water deficit effect on activities of SuS*as*, AGP*ase,* SSS*ase* in superior (S) and inferior (I) grains of wheat.

Treatment		14 DPA						21 DPA					
		SuS*ase* (mg sucrose g^−1^FW min^−1^)		AGP*ase* (mol g^−1^ FW min^−1^)		SSS*ase* (mol g^−1^ FW min^−1^)		SuS*ase* (mg sucrose g^−1^FW min^−1^)		AGP*ase* (mol g^−1^ FW min^−1^)		SSS*ase* (mol g^−1^ FW min^−1^)	
		S	I	S	I	S	I	S	I	S	I	S	I
WW	Control	10.6 ± 0.59ab	9.2 ± 1.24b	26.2 ± 0.92c	16.7 ± 0.28b	23.7 ± 0.41ab	11.8 ± 0.79b	10.7 ± 0.14bc	8.3 ± 0.58bc	16.7 ± 0.8d	11.9 ± 0.9d	18.6 ± 0.22ab	6.1 ± 0.6bc
	Spd	12.5 ± 0.36ab	6.4 ± 0.05c	34.4 ± 1.8ab	23.37 ± 0.19ab	17.3 ± 0.25d	14.3 ± 0.7ab	13.5 ± 0.67b	8.4 ± 1.58bc	23.3 ± 1.3c	18.9 ± 0.52ab	19.7 ± 0.17a	8.3 ± 0.45ab
	AVG	11.5 ± 0.21ab	7.9 ± 0.41bc	28.1 ± 1.7bc	31.1 ± 1.1a	19.5 ± 0.64 cd	13.7 ± 0.36ab	18.5 ± 0.54a	13.9 ± 0.49a	31.1 ± 2.2a	15.2 ± 0.71bc	17.3 ± 0.29b	9.1 ± 0.3a
SD	Control	7.5 ± 0.2b	6.6 ± 0.57c	23.3 ± 0.4c	16.3 ± 0.56b	20.9 ± 1.04bc	5.8 ± 0.11c	7.1 ± 0.1c	4.6 ± 0.42c	16.3 ± 0.19d	8.7 ± 0.99e	16.3 ± 0.86bc	2.2 ± 0.18d
	Spd	14.9 ± 0.47ab	13.3 ± 0.47a	36.8 ± 1.5a	32.8 ± 0.32a	25.4 ± 1.25a	12.7 ± 0.87b	11.7 ± 1.13b	4.9 ± 0.24c	26.9 ± 0.35b	18.22 ± 0.25a	20.7 ± 0.09a	3.35 ± 0.55 cd
	AVG	16.3 ± 0.21a	9.9 ± 0.34b	28.3 ± 0.39bc	26.9 ± 0.12a	18.1 ± 0.12 cd	16.1 ± 1.22a	18.0 ± 1.21a	12.1 ± 1.26ab	32.77 ± 0.62a	12.8 ± 0.19 cd	14.1 ± 0.08bc	7.6 ± 0.29ab
P-value	Water treatment (W)	0.3096	0.0002	0.9531	0.3617	0.0402	0.0069	0.0183	0.0013	0.0149	0.0116	0.0036	0.0001
	Chemical treatment (C)	0.0184	0.005	0.0001	0.0002	0.0014	0.0001	0.0001	0.0001	0.0001	0.0001	0.0001	0.0001
	W × C	0.0796	0.0001	0.2469	0.0206	0.0001	0.0003	0.2197	0.4898	0.0453	0.006	0.004	0.0575

Means (*n = *3) within a column with the same letter are not significantly different at *P* < 0.05. WW, well-watered; SD, severe water deficit; AVG, Aminoethoxyvinylglycine; Spd, Spermidine;

S, superior grains; I, inferior grains.
