# Supplementary material for: Involvement of ethylene and polyamines biosynthesis and abdominal phloem tissues characters of wheat caryopsis during grain filling under stress conditions
Source: Sci Rep. 2017 Apr 6;7:46020. doi: 10.1038/srep46020 (PMC5382545; doi:10.1038/srep46020)
Supplement: Supplementary Information [file srep46020-s1.pdf]

Supplementary information

Involvement of ethylene and polyamines biosynthesis and abdominal phloem tissues characters of wheat caryopsis during grain filling under stress conditions

Weibing Yang[1,2]*, Yanxia Li[1]*, Yanping Yin[1]*, Zhilie Qin[2], Mengjing Zheng[1], Jin Chen[1], Yongli Luo[1], Dangwei Pang[1], Wenwen Jiang[1], Yong Li[1], Zhenlin Wang[1]

[1] State Key Laboratory of Crop Biology, Ministry of Science and Technology, Shandong Agricultural University, Tai'an 271018, Shandong, China.

[2] Beijing Academy of Agricultural and Forestry Sciences, Beijing Engineering Research Center for Hybrid Wheat. Beijing, 100097, China.

* These authors contributed equally to this study

Correspondence and requests for materials should be addressed to Y.L. (e-mail: xmliyong@sdau.edu.cn) or Z.W. (e-mail: zlwang@sdau.edu.cn)

Supplementary Methods

Starch assays. The contents of amylose, amylopectin and starch were determined according to the reports by Schaffer and Petreikov[1] with modifications. 0.1 g of milled grains were stirred with 10 ml of 0.5 M KOH for 15 min at 35℃ and then diluted to a volume of 50 ml with distilled water. Of this solution, 2.5 ml were diluted with 20 ml distilled water and adjusted to pH 3.5 with 0.1 M HCl, and then 0.5 ml of I_2-KI reagent was added to the solution, which was diluted with distilled water to a final volume of 50 ml. After blending for 20 min, the mixture was monitored with a UV-160A Shimadzu spectrophotometer at 461.5, 555, 656.5 and 760 nm. The absorption peaks of purified amylose reacted with I_2-KI reagent were 656.5 and 461.5 nm, whereas those of amylopectin were 760 and 555 nm. A 0.1 g of dried grain sample was extracted with 80% ethanol (V/V) in 80 ℃ water bath. The contents of sucrose were determined using the anthrone method.

Enzyme extraction and enzyme activity testing. Enzyme extraction was according to the method by Ranwala[2] and the enzyme activity testing was according to the method described by Nakamura[3]. 20 to 30 frozen grains were weighed and homogenised with a pestle in an ice-cold motor, which contained 8 ml of 50 mM Hepes–NaOH (pH 7.5), 10 mM MgCl2, 2 mM EDTA, 50 mM 2-mercaptoethanol, 12.5 % (v/v) glycerol, and5 % (w/v) insoluble PVP (polyvinylpyrrolidone-40). To 30 μl of the homogenate was added 1.8 ml of the buffer solution, which was then centrifuged at 2000 g at 0–4 ℃. The rest of the homogenate was centrifuged at 10 000 g at 0-4 °C for 10 min for activities of SuSase, AGPase, and SSSase assay. All enzymes were not purified to avoid enzyme activity loss. During assay, however, the background value of each sample was determined by adding the same volume of denatured enzyme extraction to correct the possible substrate in the crude enzyme extraction.

SuSase

The reaction mixture contained 50 mM Hepes–NaOH (pH 7.5), 50 mM fructose, 100 mM UDPglucose, 50 mM $MgCl_2$ and made up a volume of 3.5 ml. The reaction was started by adding 0.2 ml of enzyme crude extraction. After 30 min, the reaction was terminated in boiling water for 1 min. Then 0.2 ml of 2 M NaOH was added to the solution, which was heated for 10 min in boiling water. After cooling, 2 ml of 30 % HCl and 1 ml of 0.1 % resorcin were added to the solution, which was then heated for 10 min in 80 °C water. Finally, the formation of UDP glucose-dependent sucrose catalyzed by SuS was monitored at 480 nm with a UV-160A Shimadzu spectrophotometer.

AGPase

The reaction solution contained 100 μl of 1.2 mM ADPG (UDPG), 50 μl of 6 mM $MgCl_2$, 400 μl of 50 mM Hepes–NaOH (pH 7.5), and 50 μl (ADPG) or 20 μl (UDPG) of enzyme crude extraction. After incubated at 30 °C for 10 min, the reaction was initiated by adding 100 μl of 5 mM PPi and stopped after 15 min by heating at boiling water. After cooling, 100 μl of 6 mM NADP+, 1.5 IU phosphoglucomutase (EC5.4.2.2), 1 IU 6-phosphogluconate dehydrogenase (EC1.1.1.44), 5 IU glucose-6-phosphate dehydrogenase (EC1.1.1.49), 50 μl of 6 mM $MgCl_2$, and 200 μl of 50 mM Hepes–NaOH (pH 7.5) were added to the reaction solution. The mixture was incubated at 30 °C for 10 min, and then monitored at 340 nm.

SSSase

The reaction solution contained 100 μl of 1.6 mM ADPG and 700 μl of 50 mg/ml amylopectin. After incubation at 3 °C for 5 min, the reaction was initiated by adding 50 μl enzyme extraction and stopped after 20 min by heating in boiling water. The ADP produced by the SSS was converted to ATP by adding 100 μl of 4 mM PEP, 50 μl of 20 mM $MgCl_2$ and 1.2 IU pyruvate kinase (EC2.7.1.40) and then incubating at 30 °C for 30 min. The resultant ATP was determined by adding 5 ml of

luciferin–luciferase reagent. 5 ml of luciferin–luciferase reagent were added to 50 μl enzyme extraction for measurement of ATP content.

RNA extraction, cDNA biosynthesis, and quantitative real-time PCR. Genes encoding enzymes involved in PA biosynthesis**,** *ADC1, ADC2, ODC***,** *Agmatinase***,** *SAMDC, Spd1* and *Spd2, and in* ethylene biosynthesis, ***ACS****, and the* sucrose transporter, *TaSUT1,* were analysed at the transcript level. These genes were chosen because they were observed to be closely associated with grain filling of wheat in our previous studies. RNA extraction, cDNA preparation, and real-time fluorescence quantitative PCR (qRT-PCR) were carried out using the method described by Chen[39] with minor modifications. Total RNA in grains was extracted with the RNeasy plant mini kit (Qiagen, Gemerny) following the manufacturer's protocols, and was isolated and transcribed with oligo (dT) primers using a SuperScript first-strand synthesis system according to the manufacturer's instructions (Invitrogen, USA). Transcript levels of the genes were measured by qRT-PCR using an ABI-7700 with SYBR GreenI (ABI, USA). The gene-specific primer pairs used for qRT-PCR are listed as follows. The amplification of the Actin gene was performed as a control, and a standard curve for each gene was checked to calculate the transcript levels. Three replications were conducted for each sample.

References

1. Schaffer, A. A.& Petreikov, M. Sucrose-to-starch metabolism in tomato fruit undergoing transient starch accumulation. Plant Physiol. **113,** 739–746 (1997).

2. Ranwala A. P. & Miller W. B. Sucrose-cleaving enzymes and carbohydrate pools in Lilium longiflorum floral organs. Physiol Plant. **103,** 541–550 (1998).

3. Nakamura, Y., Yu, k. K. & Park, S.Y. Carbohydrate metabolism in the developing endosperm of rice grains. Plant Cell Physiol. **30,** 833–839 (1989).
